# Targeting collagen in tumor extracellular matrix as a novel targeted strategy in cancer immunotherapy

**DOI:** 10.3389/fonc.2023.1225483

**Published:** 2023-08-24

**Authors:** Jiayang Liu, Danjie Pan, Xuan Huang, Songna Wang, Huaning Chen, Yi Zhun Zhu, Li Ye

**Affiliations:** ^1^ Department of Biological Medicines at School of Pharmacy, Minhang Hospital, Fudan University, Shanghai, China; ^2^ Shanghai Engineering Research Center of Immunotherapeutics, School of Pharmacy, Fudan University, Shanghai, China; ^3^ School of Pharmacy and State Key Laboratory of Quality Research in Chinese Medicine, Macau University of Science and Technology, Macau, Macau SAR, China

**Keywords:** collagen-binding domain, collagen-binding protein, tumor targeting, cancer immunotherapy, collagen

## Abstract

Collagen, the most abundant protein in mammal, is widely expressed in tissues and organs, as well as tumor extracellular matrix. Tumor collagen mainly accumulates in tumor stroma or beneath tumor blood vessel endothelium, and is exposed due to the fragmentary structure of tumor blood vessels. Through the blood vessels with enhanced permeability and retention (EPR) effect, collagen-binding macromolecules could easily bind to tumor collagen and accumulate within tumor, supporting tumor collagen to be a potential tumor-specific target. Recently, numerous studies have verified that targeting collagen within tumor extracellular matrix (TEM) would enhance the accumulation and retention of immunotherapy drugs at tumor, significantly improving their anti-tumor efficacy, as well as avoiding severe adverse effects. In this review, we would summarize the known collagen-binding domains (CBD) or proteins (CBP), their mechanism and application in tumor-targeting immunotherapy, and look forward to future development.

## 1 Introduction

Cancer immunotherapy has risen rapidly since IL-2 was approved by FDA in 1991 for cancer immunotherapy, which significantly improved the prognosis of patients with multiple metastatic or refractory tumor in the following decades. Quite a few immunotherapy candidates have demonstrated inspiring outcomes in reducing recurrence rates after surgery, radiotherapy or chemotherapy and prolonging the survival of patients in clinical trials (1–4). However, only a fraction of candidates could be finally approved. One of the obstacles is side effect, which is regarded as the most common but vital problem in immunotherapy drugs development (5, 6). Most side effects of immunotherapy drugs occurs because of binding to targets in non-tumor tissues, or over-activating peripheral immune system (7), which indicating that effective responses are not properly controlled. Thus, tumor-targeting ability seems to be a solution for those agents, which would focus drug function on tumor and reverse off-target side effects. For this, a tumor-specific target is supposed to be necessary.

Collagen is the most abundant protein in mammals, widely existing in tissues and organs (8), especially tumor (9). When growing rapidly, tumor forms abnormal blood vessels with fragmentary structure, which expose tumor collagen beneath to blood. The abnormal tumor blood vessels also attract and retain macromolecules in blood, which is summarized as the enhanced permeability and retention (EPR) effect. Thus, collagen-binding macromolecules would accumulate within tumors rather than other tissues, because of different permeability. The unique feature in tumor supported collagen to be an ideal tumor-specific target, which has been verified and reported in a mass of researches.

## 2 Collagen in tumor

Collagen family consists of 28 types of collagens named from collagen type I to XXVIII, which were successively discovered since 1969, when collagen type II was identified by Miller and Matukas (10). Collagen is composed of three α-chains with a triple helix domain and sometimes two non-helical domains at each side, while different α-chain subtype combinations form different collagens (11). The mutation or abnormal overexpression of collagen have been verified to be highly associated with various diseases such as osteoporosis, achondroplasia, Ehlers-Danlos syndrome, Alport syndrome, and cancer (12, 13). Tumor collagen mainly exists in tumor stroma or beneath tumor blood vessel endothelium, promoting angiogenesis and progression of tumor, as well as supporting invasion and metastasis of cancer cells (9). Tumor collagen along with other components in tumor extracellular matrix also help inhibit anti-tumor immune response via preventing the infiltration of immune cells, which vastly depresses the effect of immunotherapy drugs and leads to poor prognosis (14, 15). Constitutively expressed in almost all types of solid tumors, collagen is supposed to become a potential anti-tumor target. Quite a few immunotherapies targeting tumor collagen have represented significant anti-tumor efficacy through regulating the expression or alignment of collagen (16–19).

In order to meet the terrible desires for oxygen and nutrition, tumor upregulates the expression of vascular endothelial growth factor (VEGF) and reduces inhibitory factors such as thrombospondin and angiostatins (20), forming plenty of abnormal blood vessels with irregular shapes, multiple twists and blind ends, which are easy to leak and bleed (21). In a widely accepted theory, tumor is regarded as incurable wound, with numerous similar characteristics such as high level of VEGF-A expression, highly permeable sinusoids called “mother vessels” at the beginning of angiogenesis, and a large number of blood capillaries linking mother vessels (21, 22). The over-expressed VEGF-A and its subtypes which come from alternative splicing effectively promote angiogenesis and vascular permeability, leading to the abnormity of tumor blood vessels (22). Meanwhile, some other vascular permeability factors such as bradykinin and nitric oxide are also upregulated within tumor, collectively contributing to the EPR effect of tumor (23–25).

**Figure 1:**
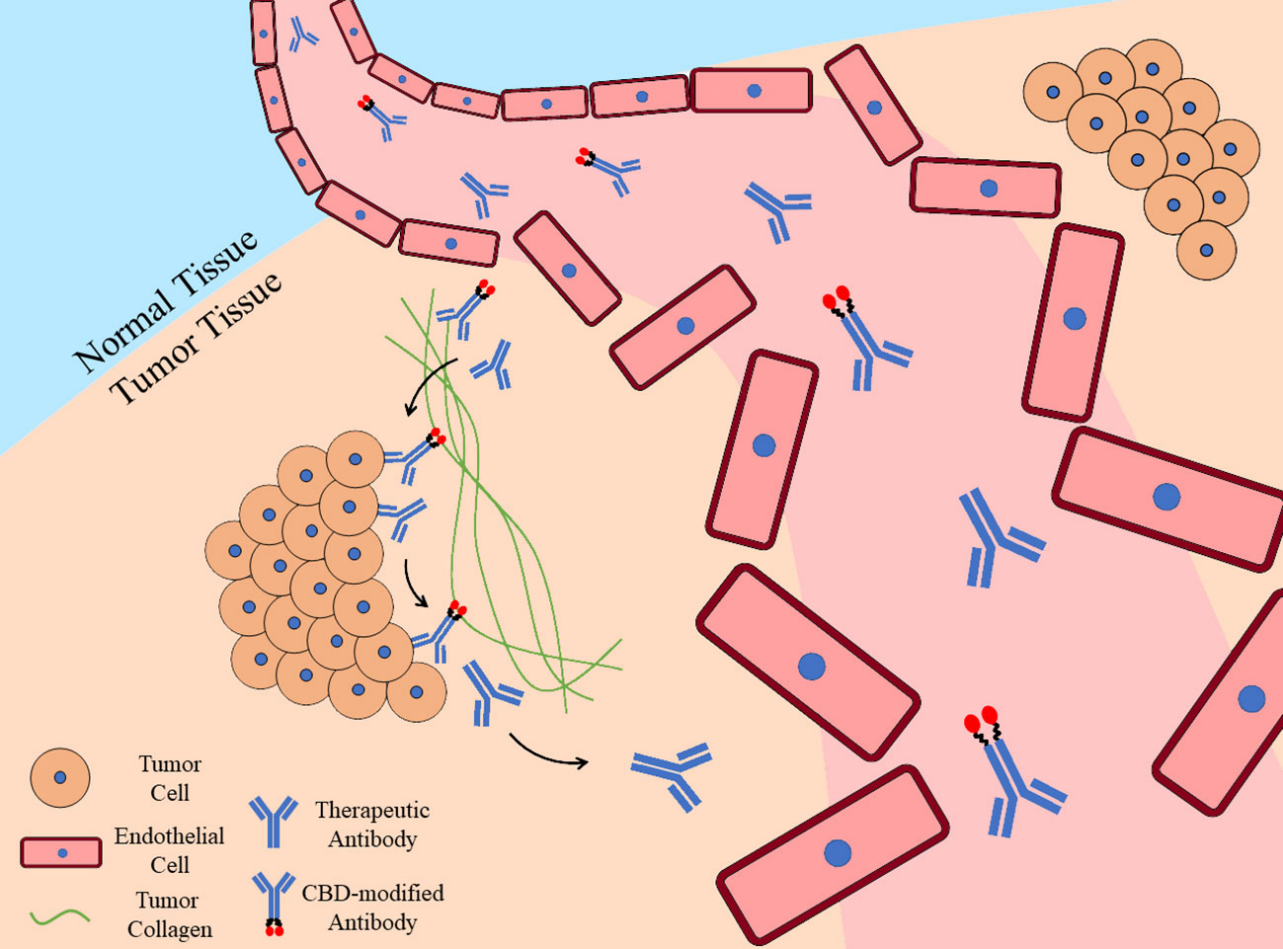
Tumor-targeting ability and prolonged retention of CBD-modified antibody. Once therapeutic antibodies enter tumor tissues due to the fragmentary tumor blood vessels, they would bind specific targets on tumor cells, while CBD-modified antibodies would also bind to tumor collagen with their collagen-binding domains. Benefited from the collagen affinity, CBD-modified antibodies would retain in tumor for longer time while unmodified ones are eliminated.

EPR effect is widely used in tumor targeting strategies, including strategy of targeting tumor collagen. Benefited from EPR effect, collagen-binding macromolecules would enter tumor tissues rather than other organs when circulating in blood and bind exposed collagen around tumor blood vessels. When linked to immunotherapy drugs such as antibodies or therapeutic cytokines, collagen-binding macromolecules would represent tumor-targeting ability and prolong the retention of drugs within tumor (15) (**Figure 1**).

## 3 Collagen-binding domains

Collagen-binding domain (CBD) is a kind of protein domains or polypeptides that specifically bind to collagen. CBD is derived or designed from collagen-binding sites in natural ligands of collagen, such as fibronectin (FN), von Willebrand factor (vWF), placental growth factor (PlGF) and collagenase (reviewed in Addi et al., 2017 (26)).

### 3.1 Fibronectin

Fibronectin is a kind of non-collagenous glycoprotein, which widely exists in extracellular matrix, promoting adhesion, growth, migration and differentiation of cells through mediating interactions between cells and extracellular matrix [reviewed in Pankov et al., 2002 (27)]. FN mainly exists as dimer, while each monomer produced by alternative splicing varies from 230 to 270 kDa, consisting of several type I, II or III repetitive units. FN could bind to collagen, heparin and integrin, especially representing high affinity to collagen type I and II with *K*_D_ value from 13 to 58 nM (28, 29). FN binds to collagen with a 42 kDa binding domain, containing four type I and two type II repetitive units, which collectively contribute to the binding of FN-CBD to collagen (30). Moreover, the type II unit of FN-CBD is also discovered in other collagen-binding proteins such as mannose receptor and matrix metalloproteinase 2 (31, 32). Compared to natural collagen, FN prefers denatured collagen like gelatin (33), revealing FN may bind to the triple helix of collagen (34). FN plays important role in wound healing and human growth (35), thus FN-CBD is mainly used in researches for promoting wound healing or treating developmental defects.

### 3.2 Von Willebrand factor

Von Willebrand factor (vWF) is a kind of glycoprotein synthesized in endothelial cells and megakaryocyte, existing as polymer and participating in building basement membrane of blood vessels [reviewed in Lenting et al., 2015 (36)]. The main physiological function of vWF is mediating adhesion of blood platelets beneath blood vessel endothelium and promoting hemostasis by stabilizing coagulation factor VIII (37). VWF could bind to multiple ligands such as plasma protein, platelet receptor, integrin, collagen and heparin, with high affinity to collagen type I, III, IV and VI [reviewed in Bergmeier et al., 2012 (38)]. Mature vWF consists of 13 domains, in which A1 and A3 domains contain the main collagen-binding sites (39). The A1 domain weights 20.3 kDa, containing binding sites to collagen type IV and VI (40, 41), while the A3 domain is regarded as the main collagen-binding domain of vWF, weighting 19.3 kDa and containing binding sites to collagen type I and III (42). As a collagen-binding domain with high affinity, vWF A3-CBD is used in collagen-targeting therapies for vascular repairing, bone regeneration and tumor treatment (26, 43, 44). Beside A1 and A3 domains, some small domains in bovine and human vWF could also specifically bind collagen (45).

### 3.3 Placental growth factor

Placental growth factor (PlGF), a member of VEGF family, is mainly expressed in placenta with four subtypes of homodimer, and a few in heart, lung, liver and other tissues [reviewed in Chau et al., 2017 (46)]. PlGF is a multifunctional factor, participating in promoting angiogenesis, chondrogenesis, wound healing and tumor growth [reviewed in Dewerchin et al., 2012 (47)]. Two subtypes of PlGF, PlGF1 and PlGF2, could specifically bind to collagen type I by a 2.8 kDa collagen-binding domain with higher affinity than fibronectin, heparin and other ECM proteins (48). PlGF-CBD is found to possess high isoelectric point (pI=12.0), while polypeptides with same isoelectric point but disordered sequence represent no collagen affinity, revealing that PlGF-CBD binds to collagen through recognizing specific sequence but not electrostatic binding (48).

### 3.4 Collagen-binding peptide

Natural collagen-binding domains could bind to multiple types of collagens with high affinity. However, whole protein or large domain may induce steric hindrance and lead to difficulties in modifying cancer immunotherapy drugs. Thus, several small collagen-binding peptides were designed according to the binding site of collagen-binding proteins for better application with effective collagen-binding ability.

Heptapeptide TKKTLRT is designed according to the antisense sequence of collagenase cleavage site in mammal collagen type I α2-chain, which is also the smallest CBD peptide (49). Heptapeptide CBD is used in modifying regenerative medicine or tissue engineering drugs, such as bone morphogenetic protein-2 (BMP-2) (50) or brain-derived neurotrophic factor (BDNF) (51–53) for treatment of spinal cord injury, as well as platelet-derived growth factor (PDGF) for promoting wound healing (54). Recently, heptapeptide CBD comes to be used in researches such as modifying exosome for promoting neurogenesis in neurodegenerative disease (55) or anti-tumor therapeutic antibodies and cytokines (44, 56, 57).

Different from heptapeptide CBD, collagen mimetic peptide (CMP) is designed imitating the triple helix in collagen, which could bind collagen chains to form hybridized triple helix. CMP tends to bind the unfolded chain in denatured proteins, thus is considered to be a potential material in diseases with collagen degeneration or degradation such as atherosclerosis, osteoarthritis and fibrosis (58). In some researches, CMP is also used as hydrogel or polymeric conjugate biomaterial for supporting organs and healing wounds [reviewed in Strauss et al., 2017 and Chattopadhyay et al., 2014 (59, 60)].

## 4 Collagen-binding proteins

Apart from collagen-binding domains, some small proteins with collagen affinity are also used in collagen affinity modification, such as lumican, bacterial surface proteins and avimer.

### 4.1 Lumican

Lumican is a kind of small leucine-rich proteoglycans (SLRPs) which exists in the ECM of cornea, gristle and skin (61). Lumican regulates generation of collagenous fiber, formation of cuticle and transparency of cornea, as well as epithelial-mesenchymal transition (EMT), cell adhesion and migration in tumor progression [reviewed in Giatagana et al., 2021 (62)]. The protein fraction of lumican weights 38 kDa, consisting of four domains, with its collagen-binding site in the domain at C-terminal (63). Lumican binds to collagenous fiber to support the structure of collagen and help with wound healing in tissues like cornea [reviewed in Karamanou et al., 2018 (64)]. Recently, lumican was reported to be fused to therapeutic cytokines for cancer immunotherapy, which prolonged the retention of drugs in tumor and reduced side effects (65).

### 4.2 Bacterial surface proteins

Bacterial surface proteins mediate adhesion and invasion of bacterial and the formation of biofilm, helping bacterial to escape from host immune system (66). Several bacterial surface proteins such as lipoprotein SLR, M and M-like proteins possess collagen affinity for infection at collagen-exposed wounds (reviewed in Avilés-Reyes et al., 2017 (67)). Bacterial collagen-binding proteins could specifically bind different types of collagens, representing important values in drug design studies (67).

### 4.3 Avimer

Avimer comes from the A domain of human cell membrane proteins which mediates interaction between proteins. There are more than 200 kinds of avimers, most of which consist of 35 amino acids and weight about 4 kDa [reviewed in Weidle et al., 2013 (68)]. In a recent study, avimers with high collagen affinity were selected through phage display technology and fused it to IL-1 for treatment of joint diseases (69).

## 5 Cancer immunotherapy targeting tumor collagen

**Figure 2:**
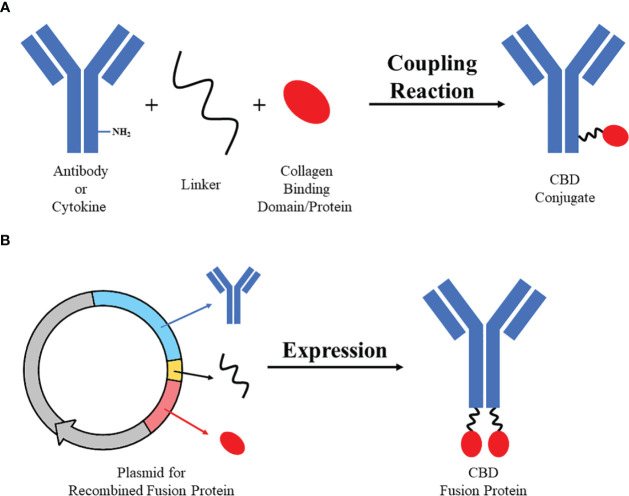
Two strategies of designing collagen-binding drugs. Strategy **(A)** Collagen-binding domain/protein with active group (e.g. sulfydryl) could be bound to active group on antibodies or cytokines (e.g. amino) by specific linker (e.g. SMCC) through simple coupling reaction to synthesize CBD conjugate. Strategy **(B)** Sequences of antibodies/cytokines, linker and collagen-binding domain/protein could be recombined and expressed through eukaryotic expression system to produce CBD fusion protein.

Collagen-targeting drugs are mostly based on two strategies: one is linking collagen-binding domains or collagen-binding proteins to drugs at active chemical groups to produce collagen-binding conjugates, the other is designing therapeutic fusion proteins which contain CBD or CBP (44) (**Figure 2**). Collagen-binding drugs used to be studied for diseases at collagen-rich tissues or wounds (26, 60, 69, 70). Recently, more and more researches on CBD or CBP-modified cancer immunotherapeutic drugs raised (56, 57, 65).

A variety of immunotherapy drugs have been modified with CBD or CBP, including therapeutic antibodies such as anti-EGFR antibody (56, 57), anti-PDL1 antibody and anti-CTLA4 antibody (44), and cytokines such as IL-2 (44) and IL-12 (65, 71). Hui Liang and colleagues successively reported collagen-binding EGFR single-chain Fv (scFv) antibody (56) and EGFR Fab antibody (57), which fused antibody fragment to heptapeptide CBD TKKTLRT. In their studies, both CBD-scFv and CBD-Fab retained EGFR affinity and anti-tumor activity, while CBD contributed to an development of collagen affinity, which led to quicker accumulation, longer retention and controlled release of drugs in tumor, representing enhanced anti-tumor efficacy on nude mice A431 xenograft model (56, 57). In 2019, Jun Ishihara and colleagues also applied heptapeptide CBD on modification of CTLA4 and PDL1 antibodies (44). Apart from advantages such as collagen affinity, enhanced accumulation and anti-tumor efficacy that similar to previous studies, they reported reduced treatment-related toxicity of CBD modification, including water content in liver, increase of alanine aminotransferase (ALT) and aspartate aminotransferase (AST) activity and other organs damage markers in C57BL/6 B16F10 allograft mouse model (44), supporting CBD to be an potential solution for adverse effects induced by therapeutic antibodies. Previously, our group reported a CBD-SIRPαFc conjugate as a novel tumor-targeting CD47 inhibitor (72). We conjugated collagen-binding domain TKKTLRT to SIRPαFc fusion protein with a short chemical linker Sulfo-SMCC, which could block immune checkpoint CD47 and promote anti-tumor phagocytosis to suppress tumor growth. CBD-SIRPαFc derived collagen affinity and showed faster accumulation and prolonged retention in tumor than unmodified SIRPαFc, providing a possible strategy to avoid off-target adverse reactions in anti-CD47 therapy. Hence, CBD-SIRPαFc represented improved anti-tumor efficacy with increased macrophage infiltration and activation compared to unmodified SIRPαFc on nude mice A549 xenograft model.

Collagen-binding cytokines benefit more from CBD or CBP because of their common peripheric side effects. IL-2 and IL-12 could activate T cells and NK cells, promote antigen presentation and secretion of IFN-γ (44, 73). However, due to the severe immune-related adverse effects (irAEs) including pulmonary edema, hematologic toxicity and death by systemic administration (44, 74), as well as poor accumulation in tumor (75), therapeutic cytokines are limited in application. Jun Ishihara and colleagues reported a fusion protein of IL-2 and vWF A3-CBD, which reduced splenomegaly and pulmonary edema induced by IL-2-related vascular leakage and improved its anti-tumor efficacy in C57BL/6 B16F10 allograft mouse model (44). Besides, Noor Momin and colleagues reported two fusion proteins of collagen-binding protein lumican with IL-2 and IL-12, which represented impressive anti-tumor efficacy when administrated individually or jointly in multiple mouse models (65). Lumican-IL-2 and lumican-IL-12 also enhanced combined therapies such as therapeutic antibodies, cancer vaccines and CAR-T therapy without systemic toxicity (65). In 2020, Aslan Mansurov reported a vWF A3 CBD-IL-12 fusion protein, which was also improved with better anti-tumor effect and fewer toxicities in C57BL/6 B16F10 and EMT6 allograft mouse models (71). A lot of CBD or CBP-modified antibodies and cytokines had been proved their advantages in multiple tumor models with vastly different collagen density from <3% in B16F10 to 20% in 4T1 (**Table 1**) (65), supporting collagen-binding domain/protein to be a hopeful tumor-targeting strategy for improving therapeutic effect of therapeutic cytokines and reducing their common adverse effects (73).

**Table 1:** Summary of applications targeting tumor collagen.

Modified Drug	CBD or CBP used	Modification Method	Tested Model	Advantages	Reference Number
Anti-EGFR scFv	Heptapeptide TKKTLRT	Fusion Protein	Nude Mice A431 Xenograft Model	Collagen Affinity; Prolonged Retention; Controlled Release; Enhanced Anti-Tumor Efficacy	(56)
Anti-EGFR Fab	Heptapeptide TKKTLRT	Fusion Protein	Nude Mice A431 Xenograft Model	Collagen Affinity; Prolonged Retention; Controlled Release; Enhanced Anti-Tumor Efficacy	(57)
Anti-CTLA4 Antibody + Anti-PDL1 Antibody (Combination therapy)	vWF A3 Domain	Conjugate	C57BL/6 B16F10 Allograft Model	Collagen Affinity; Quicker Accumulation; Prolonged Retention; Enhanced Anti-Tumor Efficacy; Reduced Toxicity	(44)
SIRPαFc Fusion Protein	Heptapeptide TKKTLRT	Conjugate	Nude Mice A549 Xenograft Model	Collagen Affinity; Quicker Accumulation; Prolonged Retention; Enhanced Anti-Tumor Efficacy; Promoted Anti-Tumor Immune Response	(72)
IL-2	vWF A3 Domain	Fusion Protein	C57BL/6 B16F10 Allograft Model	Collagen Affinity; Quicker Accumulation; Prolonged Retention; Enhanced Anti-Tumor Efficacy; Reduced Toxicity	(44)
IL-2	Lumican	Fusion Protein	C57BL/6 B16F10, EMT6 and MC38 Allograft Model	Collagen Affinity; Quicker Accumulation; Prolonged Retention; Enhanced Anti-Tumor Efficacy; Reduced Toxicity; Potentiation of Other Therapies	(65)
IL-12	Lumican	Fusion Protein	C57BL/6 B16F10, EMT6 and MC38 Allograft Model; Balb/C 4T1 Allograft Model	Collagen Affinity; Quicker Accumulation; Prolonged Retention; Enhanced Anti-Tumor Efficacy; Reduced Toxicity; Potentiation of Other Therapies	(65)
IL-12	vWF A3 Domain	vWF A3 Domain	C57BL/6 B16F10 and EMT6 Allograft Model	Collagen Affinity; Quicker Accumulation; Prolonged Retention; Enhanced Anti-Tumor Efficacy; Reduced Toxicity; Potentiation of Other Therapies	(71)

Applications of targeting tumor collagen mentioned above are summaried with their prototype drugs, CBD or CBP used for modification, modification method, tested models and advantages. Corresponding reference numbers are also labeled.

## 6 Discussion

The growth of tumor relies on sufficient oxygen and nutrition. Thus, tumor accelerates angiogenesis and forms lots of blood vessels. However, the forced blood vessels are abnormal and highly permeable, exposing tumor collagen to drugs in circulation as a tumor-specific target. Due to the wide distribution of collagen, numerous proteins have been found to possess collagen affinity, which could be used to confer drugs with collagen-binding ability. Collagen-binding cancer immunotherapy drugs have been proved to derive tumor-targeting ability, representing enhanced accumulation and prolonged retention in tumor, as well as improved anti-tumor efficacy. Lots of immunotherapy drugs have also benefited from tumor collagen-targeting strategy with less accumulation in normal tissues and fewer peripheric adverse effects, thus could be better applied.

As an important component in tumor extracellular matrix, collagen tends to be overexpressed in most solid tumors (76), with stable content and structure among different tumor types. Though collagen is also abundant in normal tissues, the intact blood vessel walls would prevent collagen-binding macromolecules from leaking (77). CBD or CBP modified drugs tend to enter tumor through the fragmentary tumor blood vessels and bind tumor collagen, but not normal tissues, which has been repeatedly verified on mouse models in multiple researches (44, 57, 65), ensuring the safety and specificity of targeting tumor collagen. Studies on the differences between collagen in tumor and normal tissues would also contribute to the development of targeting tumor collagen, such as the overexpression (76, 78) and enhanced linearization (19) of tumor collagen. A recent study also proposed different trimer of collagen α-chains in pancreatic cancer and normal tissues (79). Such differences are being gradually discovered, which would help us distinguish tumor collagen and design tumor-specific collagen binding domains as a developed method of targeting tumor collagen in the future.

Meanwhile, collagen-binding modification is not restricted to specific drugs. Through fusion expression, various kinds of immunotherapeutic drugs could be included in targeting tumor collagen. Thus, targeting tumor collagen could be applied on not only broad spectrum of tumors but also diverse immunotherapy drugs.

Though it is a hopeful strategy, most applications of targeting tumor collagen still stay at preclinical phase, which is main limitation in this field. We are paying attention and looking forward to the outcomes of targeting tumor collagen in clinical trials, and we hope it will provide us more choices and possibilities for cancer immunotherapy.

## Author contributions

JL, DP and LY contributed to the design and writing of manuscript. XH, SW, HC and YZ contributed their opinions to the revision of manuscript. All authors contributed to the article and approved the submitted version.
